# A monoclinic polymorph of 5-[(1*H*-benzimidazol-1-yl)meth­yl]benzene-1,3-dicarb­oxy­lic acid

**DOI:** 10.1107/S1600536812041025

**Published:** 2012-10-06

**Authors:** Hai-Wei Kuai

**Affiliations:** a Faculty of Life Science and Chemical Engineering, Huaiyin Institute of Technology, Huaian 223003, People’s Republic of China

## Abstract

Crystals of the title compound, C_16_H_12_N_2_O_4_, were obtained accidentally by the hydro­thermal reaction of 5-[(1*H*-benzo[*d*]imidazol-1-yl)meth­yl]isophthalic acid with manganese chloride tetra­hydrate in the presence of KOH as alkaline reagent for the deprotonation. A triclinic polymorph of this structure has been reported previously from a similar reaction [Cheng (2011 ▶). *Acta Cryst.* E**67**, o3299]. The benzimidazole ring system is almost planar, with a maximum deviation from the mean plane of 0.020 (4) Å. The benzimidazole unit and benzene ring are inclined at a dihedral angle of 68.17 (4)°, reflecting the axial rotation of the flexible benzimidazolyl arm. In the crystal, pairs of O—H⋯O hydrogen bonds link adjacent mol­ecules into inversion dimers. O—H⋯N contacts connect these dimers into zigzag chains along [010].

## Related literature

For a triclinic polymorph of the title compound, see: Cheng (2011 ▶).
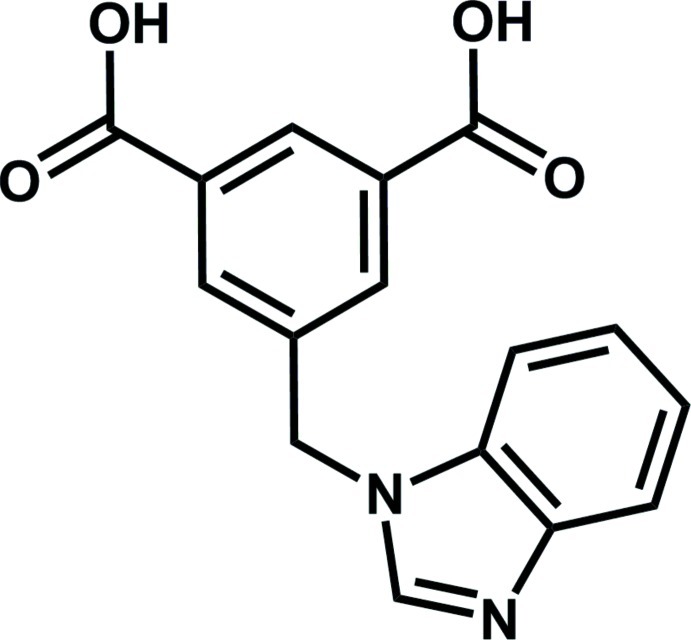


## Experimental

### 

#### Crystal data

C_16_H_12_N_2_O_4_
*M* *_r_* = 296.28
Monoclinic, 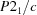

*a* = 7.401 (5) Å
*b* = 16.589 (5) Å
*c* = 11.762 (4) Å
β = 111.53 (3)°
*V* = 1343.3 (11) Å^3^
*Z* = 4
Mo *K*α radiation
μ = 0.11 mm^−1^
*T* = 293 K
0.20 × 0.10 × 0.10 mm

#### Data collection

Bruker Smart APEXII CCD diffractometer
Absorption correction: multi-scan (*SADABS*; Sheldrick, 1996 ▶) *T*_min_ = 0.979, *T*_max_ = 0.989
6722 measured reflections
2358 independent reflections
1239 reflections with *I* > 2σ(*I*)
*R*_int_ = 0.072

#### Refinement

*R*[*F* ^2^ > 2σ(*F* ^2^)] = 0.056
*wR*(*F* ^2^) = 0.152
*S* = 0.96
2358 reflections
200 parameters
H-atom parameters constrained
Δρ_max_ = 0.21 e Å^−3^
Δρ_min_ = −0.22 e Å^−3^

### 

Data collection: *APEX2* (Bruker, 2008 ▶); cell refinement: *SAINT* (Bruker, 2008 ▶); data reduction: *SAINT*; program(s) used to solve structure: *SHELXS97* (Sheldrick, 2008 ▶); program(s) used to refine structure: *SHELXL97* (Sheldrick, 2008 ▶); molecular graphics: *DIAMOND* (Brandenburg, 2000 ▶) and *Mercury* (Macrae *et al.*, 2008 ▶); software used to prepare material for publication: *SHELXTL* (Sheldrick, 2008 ▶).

## Supplementary Material

Crystal structure: contains datablock(s) I, global. DOI: 10.1107/S1600536812041025/sj5265sup1.cif

Structure factors: contains datablock(s) I. DOI: 10.1107/S1600536812041025/sj5265Isup2.hkl

Supplementary material file. DOI: 10.1107/S1600536812041025/sj5265Isup3.cdx

Supplementary material file. DOI: 10.1107/S1600536812041025/sj5265Isup4.cml

Additional supplementary materials:  crystallographic information; 3D view; checkCIF report

## Figures and Tables

**Table 1:** Hydrogen-bond geometry (Å, °)

*D*—H⋯*A*	*D*—H	H⋯*A*	*D*⋯*A*	*D*—H⋯*A*
O3—H5⋯O4^i^	0.84	1.82	2.649 (3)	171
O1—H4⋯N1^ii^	0.84	1.76	2.576 (4)	164

Symmetry codes: (i) 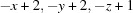; (ii) 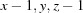.

**Fig. 1.**
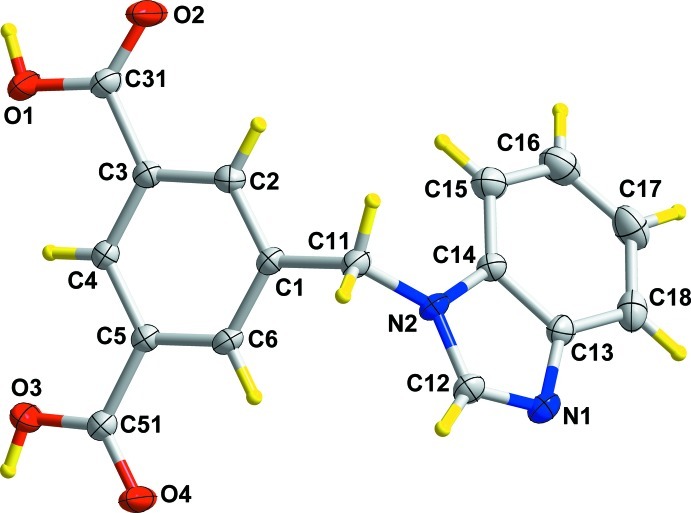
: The crystal structure of the title compound showing 30% probability displacement ellipsoids.

**Fig. 2.**
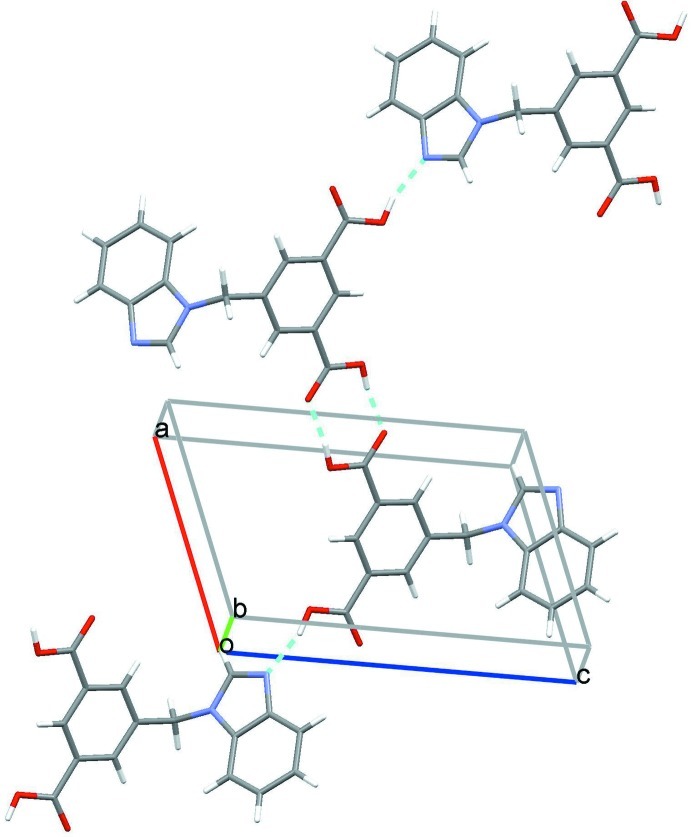
Crystal packing of the title compound.

## supplementary crystallographic information

### Comment

5-((1*H*-benzo[*d*]imidazol-1-yl)methyl)isophthalic acid (H_2_L), is usually regarded as an excellent candidate for use as a building block in molecular self-assembly engineerings due to its variable conformations and coordination modes. During an attempt to assemble a coordination polymer, we accidentally obtained some single crystals of the title compound, C_16_H_12_N_2_O_4_, as a result of the hydrothermal reaction of 5-((1*H*-benzo[*d*]imidazol-1-yl)methyl)isophthalic acid with manganese chloride tetrahydrate at 453 K in the presence of KOH as alkaline reagent for the deprotonation. A triclinic polymorph of this structure has been reported previously (Cheng, 2011) from a very similar hydrothermal reaction involving manganese bromide. The bond distances and angles in that molecule are reasonably similar to those reported here. As shown in Fig. 1, the asymmetric unit consists of only one H_2_L molecule. Interestingly, though crystallizing from alkaline solution, the H_2_L retains the intact carboxylic acid groups in the crystal structure. The flexible benzimidazolyl arm is apt to rotate axially. As a result, the benzimidazolyl ring and central benzene rings are inclined at a dihedral angle of 68.17 °.

In the crystal structure O3–H5..O4 hydrogen bonds, Table 1, link adjacent molecules into inversion dimers. O1—H4···N1 contacts connect these dimers into zig-zag chains in the 010 plane, Fig. 2.

### Experimental

A reaction mixture comprising manganese chloride tetrahydrate (23.3 mg, 0.1 mmol), 5-((1*H*-benzo[*d*]imidazol-1-yl)methyl)isophthalic acid (29.6 mg, 0.1 mmol) and KOH (11.2 mg, 0.2 mmol) in 10 ml H_2_O was sealed in a 16 ml Teflon-lined stainless steel container and heated to 453 K for 3 days. After cooling to the room temperature, colorless block like crystals of the title compound were obtained.

### Refinement

Hydrogen atoms of the OH groups were found in difference Fourier maps and their coordinates were allowed to ride on those of the O atoms with *U*_iso_(H) = 1.2*U*_eq_(O). Other hydrogen atoms were included in geometrically idealized positions and constrained to ride on their parent atoms, with C—H = 0.93–0.97 Å and *U*_iso_(H) = 1.2*U*_eq_(C).

### Crystal data

**Table:**

C_16_H_12_N_2_O_4_	*F*(000) = 616
*M**_r_* = 296.28	*D*_x_ = 1.465 Mg m^−^^3^
Monoclinic, *P*2_1_/*c*	Mo *K*α radiation, λ = 0.71073 Å
Hall symbol: -P 2ybc	Cell parameters from 479 reflections
*a* = 7.401 (5) Å	θ = 2.2–18.5°
*b* = 16.589 (5) Å	µ = 0.11 mm^−^^1^
*c* = 11.762 (4) Å	*T* = 293 K
β = 111.53 (3)°	Block, colorless
*V* = 1343.3 (11) Å^3^	0.20 × 0.10 × 0.10 mm
*Z* = 4	

### Data collection

**Table:**

Bruker Smart APEXII CCD diffractometer	2358 independent reflections
Radiation source: fine-focus sealed tube	1239 reflections with *I* > 2σ(*I*)
Graphite monochromator	*R*_int_ = 0.072
φ and ω scans	θ_max_ = 25.0°, θ_min_ = 2.2°
Absorption correction: multi-scan (*SADABS*; Sheldrick, 1996)	*h* = −8→6
*T*_min_ = 0.979, *T*_max_ = 0.989	*k* = −19→19
6722 measured reflections	*l* = −13→13

### Refinement

**Table:**

Refinement on *F*^2^	Secondary atom site location: difference Fourier map
Least-squares matrix: full	Hydrogen site location: inferred from neighbouring sites
*R*[*F*^2^ > 2σ(*F*^2^)] = 0.056	H-atom parameters constrained
*wR*(*F*^2^) = 0.152	*w* = 1/[σ^2^(*F*_o_^2^) + (0.0659*P*)^2^] where *P* = (*F*_o_^2^ + 2*F*_c_^2^)/3
*S* = 0.96	(Δ/σ)_max_ < 0.001
2358 reflections	Δρ_max_ = 0.21 e Å^−^^3^
200 parameters	Δρ_min_ = −0.22 e Å^−^^3^
0 restraints	Extinction correction: *SHELXL*, Fc^*^=kFc[1+0.001xFc^2^λ^3^/sin(2θ)]^-1/4^
Primary atom site location: structure-invariant direct methods	Extinction coefficient: 0.0052 (15)

### Special details

**Table:**

Geometry. All e.s.d.'s (except the e.s.d. in the dihedral angle between two l.s. planes) are estimated using the full covariance matrix. The cell e.s.d.'s are taken into account individually in the estimation of e.s.d.'s in distances, angles and torsion angles; correlations between e.s.d.'s in cell parameters are only used when they are defined by crystal symmetry. An approximate (isotropic) treatment of cell e.s.d.'s is used for estimating e.s.d.'s involving l.s. planes.
Refinement. Refinement of *F*^2^ against ALL reflections. The weighted *R*-factor *wR* and goodness of fit *S* are based on *F*^2^, conventional *R*-factors *R* are based on *F*, with *F* set to zero for negative *F*^2^. The threshold expression of *F*^2^ > σ(*F*^2^) is used only for calculating *R*-factors(gt) *etc*. and is not relevant to the choice of reflections for refinement. *R*-factors based on *F*^2^ are statistically about twice as large as those based on *F*, and *R*- factors based on ALL data will be even larger.

### Fractional atomic coordinates and isotropic or equivalent isotropic displacement parameters (Å^2^)

**Table:**

	*x*	*y*	*z*	*U*_iso_*/*U*_eq_	
C1	0.4929 (4)	0.81173 (19)	0.6457 (3)	0.0337 (8)	
C2	0.3157 (4)	0.81097 (19)	0.5499 (3)	0.0359 (8)	
H1	0.2128	0.7828	0.5586	0.043*	
C3	0.2869 (4)	0.85118 (19)	0.4407 (3)	0.0334 (8)	
C4	0.4398 (4)	0.89389 (18)	0.4285 (3)	0.0322 (8)	
H2	0.4224	0.9211	0.3561	0.039*	
C5	0.6190 (4)	0.89614 (19)	0.5242 (3)	0.0335 (8)	
C6	0.6444 (4)	0.85508 (19)	0.6319 (3)	0.0369 (8)	
H3	0.7645	0.8566	0.6957	0.044*	
C11	0.5228 (4)	0.7621 (2)	0.7587 (2)	0.0393 (9)	
H7	0.4032	0.7336	0.7480	0.047*	
H6	0.6222	0.7220	0.7666	0.047*	
C12	0.7566 (4)	0.8106 (2)	0.9604 (3)	0.0419 (9)	
H8	0.8643	0.7849	0.9542	0.050*	
C13	0.5734 (5)	0.87902 (19)	1.0306 (3)	0.0391 (9)	
C14	0.4574 (4)	0.85280 (19)	0.9137 (3)	0.0359 (8)	
C15	0.2615 (5)	0.8708 (2)	0.8615 (3)	0.0526 (10)	
H9	0.1855	0.8535	0.7833	0.063*	
C16	0.1854 (6)	0.9155 (2)	0.9315 (4)	0.0641 (11)	
H10	0.0545	0.9293	0.8993	0.077*	
C17	0.2977 (6)	0.9410 (2)	1.0494 (4)	0.0616 (11)	
H11	0.2394	0.9698	1.0945	0.074*	
C18	0.4931 (5)	0.9243 (2)	1.1002 (3)	0.0529 (10)	
H12	0.5688	0.9424	1.1780	0.063*	
C31	0.0929 (5)	0.8454 (2)	0.3395 (3)	0.0421 (9)	
C51	0.7854 (5)	0.9411 (2)	0.5135 (3)	0.0393 (8)	
N1	0.7629 (4)	0.85258 (17)	1.0568 (2)	0.0451 (8)	
N2	0.5798 (3)	0.80875 (16)	0.8720 (2)	0.0363 (7)	
O1	0.0865 (3)	0.87513 (17)	0.23681 (19)	0.0661 (8)	
H4	−0.0285	0.8693	0.1880	0.079*	
O2	−0.0442 (3)	0.81489 (18)	0.3545 (2)	0.0756 (9)	
O3	0.7501 (3)	0.97884 (14)	0.41101 (19)	0.0493 (7)	
H5	0.8528	1.0018	0.4151	0.059*	
O4	0.9450 (3)	0.94199 (15)	0.5985 (2)	0.0584 (8)	

### Atomic displacement parameters (Å^2^)

**Table:**

	*U*^11^	*U*^22^	*U*^33^	*U*^12^	*U*^13^	*U*^23^
C1	0.0290 (18)	0.043 (2)	0.0258 (17)	−0.0006 (16)	0.0059 (15)	0.0014 (15)
C2	0.0313 (18)	0.041 (2)	0.0351 (18)	−0.0055 (15)	0.0113 (15)	−0.0018 (16)
C3	0.0288 (18)	0.040 (2)	0.0296 (17)	0.0005 (15)	0.0088 (14)	0.0001 (16)
C4	0.0318 (18)	0.037 (2)	0.0259 (16)	−0.0018 (15)	0.0086 (15)	−0.0012 (15)
C5	0.0295 (18)	0.040 (2)	0.0282 (17)	−0.0039 (15)	0.0077 (15)	−0.0017 (15)
C6	0.0283 (19)	0.047 (2)	0.0283 (17)	−0.0037 (16)	0.0022 (15)	−0.0040 (16)
C11	0.0355 (19)	0.046 (2)	0.0304 (17)	−0.0040 (16)	0.0052 (15)	0.0010 (17)
C12	0.033 (2)	0.054 (2)	0.0341 (19)	0.0039 (17)	0.0064 (16)	0.0062 (18)
C13	0.037 (2)	0.047 (2)	0.0307 (18)	−0.0021 (17)	0.0093 (16)	0.0076 (17)
C14	0.0298 (19)	0.044 (2)	0.0327 (18)	−0.0008 (16)	0.0096 (15)	0.0020 (16)
C15	0.040 (2)	0.067 (3)	0.049 (2)	−0.0017 (19)	0.0143 (19)	0.007 (2)
C16	0.048 (2)	0.076 (3)	0.074 (3)	0.008 (2)	0.029 (2)	0.012 (2)
C17	0.063 (3)	0.065 (3)	0.071 (3)	0.014 (2)	0.042 (2)	0.008 (2)
C18	0.070 (3)	0.054 (3)	0.039 (2)	−0.003 (2)	0.026 (2)	−0.0002 (18)
C31	0.033 (2)	0.052 (2)	0.034 (2)	−0.0013 (17)	0.0048 (17)	0.0042 (18)
C51	0.035 (2)	0.050 (2)	0.0307 (18)	−0.0049 (17)	0.0092 (17)	−0.0014 (17)
N1	0.0376 (17)	0.059 (2)	0.0304 (15)	0.0002 (14)	0.0031 (13)	0.0004 (15)
N2	0.0275 (15)	0.0483 (18)	0.0257 (14)	−0.0005 (13)	0.0011 (12)	0.0013 (13)
O1	0.0401 (15)	0.115 (2)	0.0312 (13)	−0.0222 (14)	−0.0014 (12)	0.0127 (15)
O2	0.0357 (15)	0.123 (3)	0.0534 (16)	−0.0303 (15)	−0.0005 (13)	0.0262 (16)
O3	0.0402 (14)	0.0641 (17)	0.0421 (14)	−0.0163 (12)	0.0134 (11)	0.0060 (13)
O4	0.0310 (14)	0.088 (2)	0.0449 (14)	−0.0173 (13)	0.0008 (12)	0.0130 (14)

### Geometric parameters (Å, º)

**Table:**

C1—C2	1.380 (4)	C13—N1	1.392 (4)
C1—C6	1.390 (4)	C13—C18	1.394 (4)
C1—C11	1.510 (4)	C13—C14	1.397 (4)
C2—C3	1.393 (4)	C14—C15	1.384 (4)
C2—H1	0.9300	C14—N2	1.386 (4)
C3—C4	1.386 (4)	C15—C16	1.374 (5)
C3—C31	1.494 (4)	C15—H9	0.9300
C4—C5	1.390 (4)	C16—C17	1.395 (5)
C4—H2	0.9300	C16—H10	0.9300
C5—C6	1.389 (4)	C17—C18	1.375 (5)
C5—C51	1.484 (4)	C17—H11	0.9300
C6—H3	0.9300	C18—H12	0.9300
C11—N2	1.463 (4)	C31—O2	1.203 (4)
C11—H7	0.9700	C31—O1	1.289 (4)
C11—H6	0.9700	C51—O4	1.236 (3)
C12—N1	1.317 (4)	C51—O3	1.297 (3)
C12—N2	1.340 (4)	O1—H4	0.8397
C12—H8	0.9300	O3—H5	0.8356
			
C2—C1—C6	118.4 (3)	C18—C13—C14	120.4 (3)
C2—C1—C11	120.1 (3)	C15—C14—N2	132.1 (3)
C6—C1—C11	121.4 (3)	C15—C14—C13	122.3 (3)
C1—C2—C3	121.7 (3)	N2—C14—C13	105.5 (3)
C1—C2—H1	119.1	C16—C15—C14	116.3 (3)
C3—C2—H1	119.1	C16—C15—H9	121.8
C4—C3—C2	119.1 (3)	C14—C15—H9	121.8
C4—C3—C31	122.2 (3)	C15—C16—C17	122.3 (4)
C2—C3—C31	118.8 (3)	C15—C16—H10	118.9
C3—C4—C5	120.2 (3)	C17—C16—H10	118.9
C3—C4—H2	119.9	C18—C17—C16	121.2 (4)
C5—C4—H2	119.9	C18—C17—H11	119.4
C6—C5—C4	119.6 (3)	C16—C17—H11	119.4
C6—C5—C51	119.1 (3)	C17—C18—C13	117.4 (3)
C4—C5—C51	121.3 (3)	C17—C18—H12	121.3
C5—C6—C1	121.0 (3)	C13—C18—H12	121.3
C5—C6—H3	119.5	O2—C31—O1	123.7 (3)
C1—C6—H3	119.5	O2—C31—C3	121.7 (3)
N2—C11—C1	114.4 (3)	O1—C31—C3	114.6 (3)
N2—C11—H7	108.7	O4—C51—O3	123.6 (3)
C1—C11—H7	108.7	O4—C51—C5	120.9 (3)
N2—C11—H6	108.7	O3—C51—C5	115.6 (3)
C1—C11—H6	108.7	C12—N1—C13	105.2 (3)
H7—C11—H6	107.6	C12—N2—C14	107.0 (3)
N1—C12—N2	113.4 (3)	C12—N2—C11	126.3 (3)
N1—C12—H8	123.3	C14—N2—C11	126.4 (3)
N2—C12—H8	123.3	C31—O1—H4	106.1
N1—C13—C18	130.7 (3)	C51—O3—H5	107.3
N1—C13—C14	109.0 (3)		
			
C6—C1—C2—C3	1.0 (5)	C16—C17—C18—C13	−1.7 (5)
C11—C1—C2—C3	−175.2 (3)	N1—C13—C18—C17	−179.7 (3)
C1—C2—C3—C4	−0.8 (5)	C14—C13—C18—C17	0.4 (5)
C1—C2—C3—C31	178.0 (3)	C4—C3—C31—O2	−172.8 (3)
C2—C3—C4—C5	0.1 (5)	C2—C3—C31—O2	8.5 (5)
C31—C3—C4—C5	−178.6 (3)	C4—C3—C31—O1	7.6 (5)
C3—C4—C5—C6	0.3 (4)	C2—C3—C31—O1	−171.1 (3)
C3—C4—C5—C51	179.7 (3)	C6—C5—C51—O4	0.3 (5)
C4—C5—C6—C1	−0.1 (5)	C4—C5—C51—O4	−179.2 (3)
C51—C5—C6—C1	−179.6 (3)	C6—C5—C51—O3	−179.1 (3)
C2—C1—C6—C5	−0.5 (5)	C4—C5—C51—O3	1.5 (4)
C11—C1—C6—C5	175.6 (3)	N2—C12—N1—C13	1.3 (4)
C2—C1—C11—N2	−121.4 (3)	C18—C13—N1—C12	178.4 (3)
C6—C1—C11—N2	62.6 (4)	C14—C13—N1—C12	−1.7 (4)
N1—C13—C14—C15	−179.2 (3)	N1—C12—N2—C14	−0.5 (4)
C18—C13—C14—C15	0.7 (5)	N1—C12—N2—C11	−174.7 (3)
N1—C13—C14—N2	1.4 (3)	C15—C14—N2—C12	−179.9 (3)
C18—C13—C14—N2	−178.7 (3)	C13—C14—N2—C12	−0.6 (3)
N2—C14—C15—C16	178.6 (3)	C15—C14—N2—C11	−5.7 (5)
C13—C14—C15—C16	−0.6 (5)	C13—C14—N2—C11	173.6 (3)
C14—C15—C16—C17	−0.7 (5)	C1—C11—N2—C12	−106.8 (3)
C15—C16—C17—C18	1.9 (6)	C1—C11—N2—C14	80.2 (4)

### Hydrogen-bond geometry (Å, º)

**Table:**

*D*—H···*A*	*D*—H	H···*A*	*D*···*A*	*D*—H···*A*
O3—H5···O4^i^	0.84	1.82	2.649 (3)	171
O1—H4···N1^ii^	0.84	1.76	2.576 (4)	164

Symmetry codes: (i) −*x*+2, −*y*+2, −*z*+1; (ii) *x*−1, *y*, *z*−1.

## References

[bb1] Brandenburg, K. (2000). *DIAMOND* Crystal Impact GbR, Bonn, Germany.

[bb2] Bruker (2008). *APEX2* and *SAINT* Bruker AXS Inc., Madison, wisconsin, USA.

[bb3] Cheng, X.-C. (2011). *Acta Cryst.* E**67**, o3299. 10.1107/S1600536811047416 PMC3238952 22199801

[bb4] Macrae, C. F., Bruno, I. J., Chisholm, J. A., Edgington, P. R., McCabe, P., Pidcock, E., Rodriguez-Monge, L., Taylor, R., van de Streek, J. & Wood, P. A. (2008). *J. Appl. Cryst.* **41**, 466–470.

[bb5] Sheldrick, G. M. (1996). *SADABS* University of Göttingen, Germany.

[bb6] Sheldrick, G. M. (2008). *Acta Cryst.* A**64**, 112–122. 10.1107/S0108767307043930 18156677

